# Facilitators and barriers for HIV-testing in Zambia: A systematic review of multi-level factors

**DOI:** 10.1371/journal.pone.0192327

**Published:** 2018-02-07

**Authors:** Shan Qiao, Yao Zhang, Xiaoming Li, J. Anitha Menon

**Affiliations:** 1 Department of Health Promotion, Education, and Behavior, South Carolina SmartState Center for Healthcare Quality (CHQ), Arnold School of Public Health, University of South Carolina, Columbia, SC, United States of America; 2 School of Information, Kent State University, Kent, OH, United States of America; 3 Department of Psychology, University of Zambia, Central Administration Block Great East Road Campus, Lusaka, Zambia; The Ohio State University, UNITED STATES

## Abstract

It was estimated that 1.2 million people live with HIV/AIDS in Zambia by 2015. Zambia has developed and implemented diverse programs to reduce the prevalence in the country. HIV-testing is a critical step in HIV treatment and prevention, especially among all the key populations. However, there is no systematic review so far to demonstrate the trend of HIV-testing studies in Zambia since 1990s or synthesis the key factors that associated with HIV-testing practices in the country. Therefore, this study conducted a systematic review to search all English literature published prior to November 2016 in six electronic databases and retrieved 32 articles that meet our inclusion criteria. The results indicated that higher education was a common facilitator of HIV testing, while misconception of HIV testing and the fear of negative consequences were the major barriers for using the testing services. Other factors, such as demographic characteristics, marital dynamics, partner relationship, and relationship with the health care services, also greatly affects the participants’ decision making. The findings indicated that 1) individualized strategies and comprehensive services are needed for diverse key population; 2) capacity building for healthcare providers is critical for effectively implementing the task-shifting strategy; 3) HIV testing services need to adapt to the social context of Zambia where HIV-related stigma and discrimination is still persistent and overwhelming; and 4) family-based education and intervention should involving improving gender equity.

## Introduction

HIV/AIDS continues to be one of the world’s major public health issues, with sub-Saharan Africa being the most affected region. In 2015, it was estimated that 36.7 million people were living with HIV/AIDS, of which 25.5 million were living in sub-Saharan Africa, especially in eastern and southern Africa [1]. Zambia, a landlocked country in sub-Saharan Africa, has been heavily hit by the HIV epidemic since the late 1980s. The HIV prevalence rates peaked to 28% in the late 1990s, and it declined to 13.5% in 2009 [2]. By 2015, it was estimated that 1.2 million people were living with HIV/AIDS in Zambia [3]. An HIV prevalence of 12.9% among adults aged 15–49 years old makes Zambia one of top 10 countries with the highest HIV prevalence in the world [3]. Fighting against the HIV/AIDS epidemic for over thirty years, Zambia has developed and implemented diverse programs to prevent new infections and improve HIV treatment for those infected [3]. The scale-up of HIV-testing service is one effective national strategies to halt the epidemic.

HIV-testing is a critical step in HIV treatment cascade (diagnosis, linkage to care, engagement in care, retention in care, initiation of antiretroviral therapy, and viral suppression) for all key populations. For example, identification of HIV-infected women through HIV-testing is the first step for prevention of mother-to-child transmission of HIV (PMTCT) [4]. Scale-up of pediatric counselling and testing also significantly contributes to early treatment and reduced child mortality [5]. Couple HIV testing could facilitate disclosure of HIV status in a marital relationship, promote uptake of PMTCT, and reduce loss-to-follow up of women on treatment [6]. Free antiretroviral medicine spurred the expansion of HIV-testing service in Zambia [7]. The introduction of rapid HIV antibody tests has facilitated HIV diagnosis of people worldwide, especially those in low-income countries [8]. The advanced medicine and technologies have also shaped and revolutionized the policies and practices related to HIV-testing in Zambia.

A country-wide scale up of HIV-testing service has been observed since 1998 [9]. The number of sites providing client-initiated voluntary counseling and testing (VCT) services increased from 650 in 2006 to 1689 in 2010 [10]. Along with this expansion, the Ministry of Health (MOH) and its partners have developed various strategies for diverse sub-populations including client-initiated VCT in antenatal clinics, HIV screening among inpatients in hospitals (especially TB patients) in labor wards, provider-initiated VCT in communities and home, and HIV testing service integrated with immunization programs. In accordance with task-shifting strategy recommended by WHO (World Health Organization), the Zambian government has positively engaged paraprofessionals (e.g., traditional birth attendants, influential network leaders, lay counselors and nurses) as potential testers and has provided HIV rapid testing training and monitored their performance [11].

The national-level commitments and inputs resulted in a sharp rise of reporting among people ever tested for HIV in Zambia. However, numerous factors have influenced the quality of HIV-testing service, the feasibility and acceptability of new HIV-testing approaches, and the access to HIV-testing service for various populations. Increasing literature explores the factors that affect intentions and behaviors of taking HIV-testing, as well as the delivery and quality of HIV-testing services in Zambia. Some of them focus on demographic characteristics [12, 13], some examine the family and social relations [7, 14], some investigate the structural factors such as gender inequity and education attainment [13, 15, 16]; some highlight people’s traditional health beliefs and their perceptions on testing based on existing experiences [13, 17], and some underline the issues regarding health infrastructure [14, 18, 19]. So far, there has been no systematic review to demonstrate the trend of HIV-testing studies in Zambia since the 1990s and there has been no synthesis of key socioecological factors associated with HIV-testing practices in Zambia. This systematic review aims to describe the trend of existing literature and published HIV-testing studies in Zambia, and to summarize the multi-level factors (e.g., intrapersonal, interpersonal and structural and cultural factors) that influence the HIV-testing practice for diverse populations in Zambia, with a focus on the intentions and behaviors of taking HIV-testing and the HIV-testing service.

## Methods

### Data source

This review used the Preferred Reporting Items for Systematic Reviews and Meta-Analyses (PRISMA) as guidelines. The literature search was conducted among the following six electronic databases: Academic Search Complete, CINAHL Complete, MEDLINE with Full Text databases, PsycINFO, PubMed, and Web of Science prior to November 21, 2016. The first four databases were accessed via Ebscohost, and PubMed and Web of Science were searched separately.

To capture relevant studies, the searches were performed using the following algorithm: (HIV OR “human immunodeficiency virus”) AND Zambia* in [Title].

### Inclusion criteria

The records were reviewed by the researchers to determine whether the search results met the following criteria: (1) published in peer-reviewed journals in English prior to November 2016; (2) focused on qualitative or quantitative studies of HIV testing programs; and (3) investigated the factors affecting the uptake of HIV testing in Zambia.

### Data extraction

Data was extracted and coded using structured tables containing ten defined fields. The define field incorporated the participants’ characteristics, location and setting, research design, and the effects of the programs. The participants’ characteristics included the gender and age of the sample. The research design included such fields as sample size, study design, and type of HIV-testing. The effects of the program covered the outcomes of the program, and the research findings from individual, family and social aspects. Two researchers (S.Q. and Y.Z.) worked independently to extract data from each article and then reconciled their responses to check for consistency.

## Results

**Fig 1:**
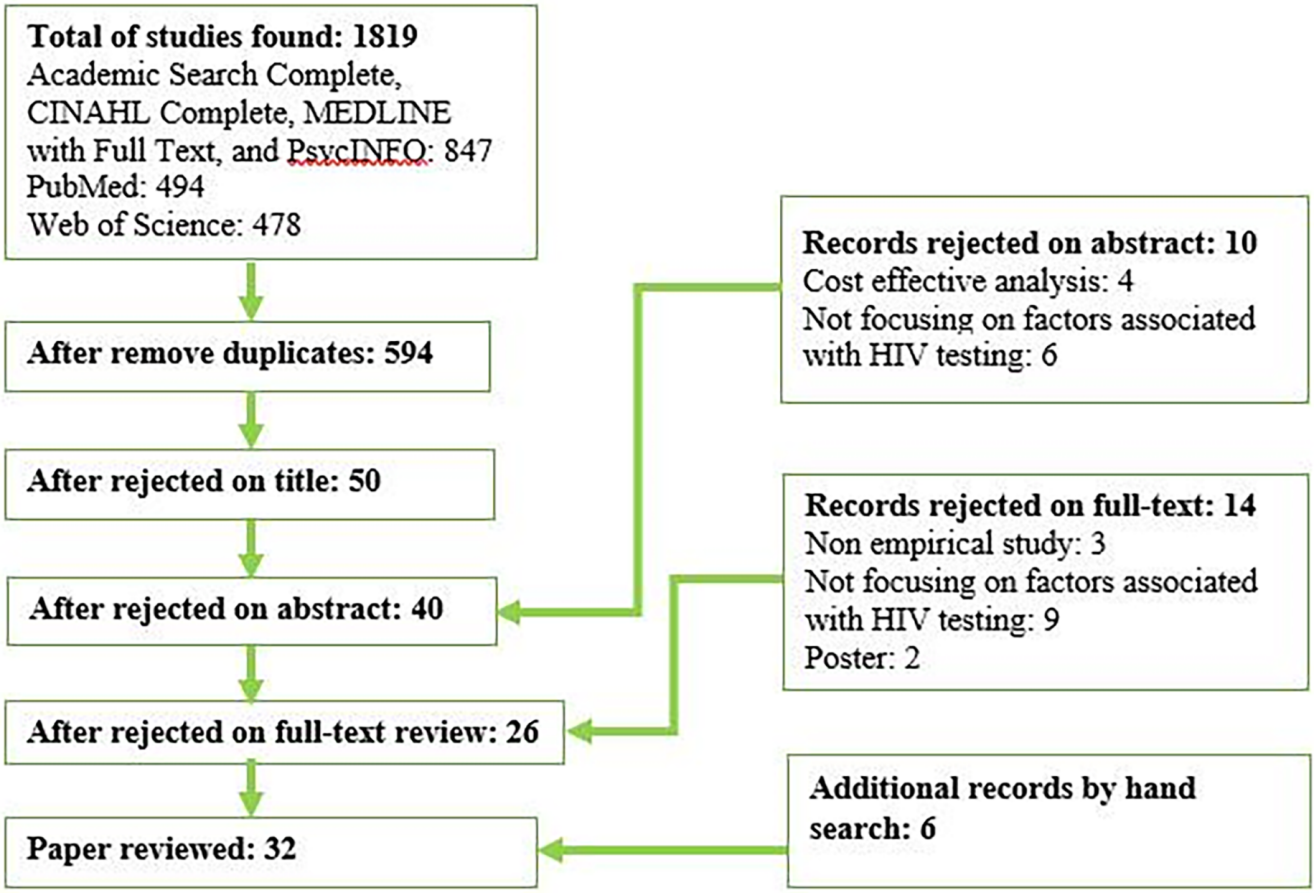
Results of literature search.

Initial searches generated 1,819 records and retained 594 records after exclusion of duplicates (Fig 1). The two researchers conducted a three-step citation screening. At the stage of title review, 544 studies were not directly relevant to HIV testing programs in Zambia, leaving 50 for further examination. During the abstract screening process, 10 additional studies were excluded because they did not fit the inclusion criteria. Four of the eleven were cost-effective analysis studies, and six did not focus on factors associated with HIV testing. Forty studies were used for full-text review, excluding 14 that did not fit the inclusion criteria. Three of the 14 exclusions were non-empirical studies, nine did not focus on factors associated with HIV testing, and two were poster presentations. A hand search was conducted within the references of the remaining articles, finding six articles that fit the inclusion criteria. A total of 32 articles were identified as eligible for inclusion.

### Characteristics of included studies

#### Research setting of the studies

**Fig 2:**
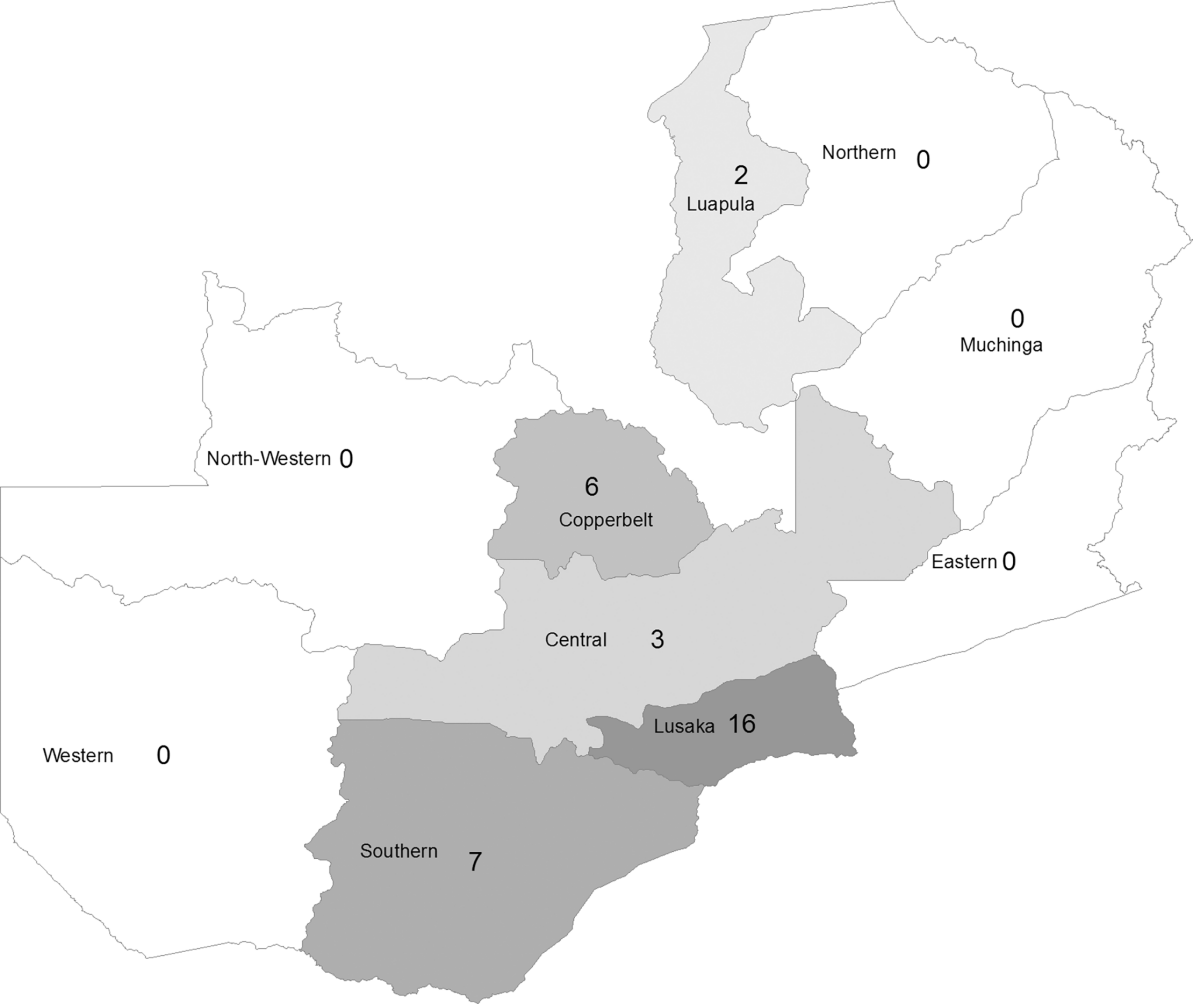
Research conducted in Zambian provinces.

The eligible articles were published between 1999 and 2016. Thirty studies provided the information of the locations where the research were conducted (Table 1). There were three studies [13, 16, 20] conducted in urban areas, four [5, 21–23] in rural areas, four [12, 16, 24, 25] in both urban and rural areas, and one [26] in rural and peri-urban area. Eleven studies [4, 18, 19, 27–34] were conducted in health facilities, such as hospitals, health centers, public sectors, VCT centers, and government clinics. The studies primarily investigated the populations in the following provinces (Fig 2): sixteen in Lusaka [4, 12–16, 23, 24, 26–29, 31, 33, 35], six in Copperbelt [7, 18–20, 30, 32], three in Central Province (including Kapiri Mposhi district) [12, 13, 24], seven in Southern Province [15, 16, 21, 22, 34, 36, 37], and two in Luapula Province [30, 32].

**Table 1:** Research setting of the studies.

#	Author and publication year	Geographic location	Year of Data Collected	Target population	Sample size	Study design	HIV testing
1	Fylkesnes et al., 1999 [12]	Chelston in urban Lusaka and Kapiri Mposhi district in rural area.	1995–1996	Urban and rural population. 15+	n = 4812	Quantitative study: Cluster randomized trial.	Voluntary counselling and testing (VCT).
2	Chi et al., 2004[27]	Birthing centers, private facilities, and the University Teaching Hospital in Lusaka. Other facilities in cities out of Lusaka.	2002	Maternity-based health care providers (physicians, midwives, and nurses).	n = 225 (physician = 78, midwife = 128, nurse = 14)	Quantitative study: Questionnaire survey. Cross-sectional study.	No detailed information provided.
3	Fylkesnes and Siziya, 2004[13]	Chelston, Lusaka and Kapiri Mposhi district in Zambia.	Baseline: 1996 Follow-up: 1999	Urban population. 15–49	n = 1886 (15–24 years of age: n = 1063 25–49 years of age: n = 823)	Quantitative study: Questionnaire survey. Longitudinal study.	Voluntary counselling and testing (VCT). Local clinics and “optional location”.
4	Thierman et al., 2006[4]	Health centers, Lusaka, Zambia	2003	Antenatal attendees 16–46	Survey: n = 1060 Focus group: n = 2 to 14 in each group	Mixed methods. Qualitative: Focus group discussion. Quantitative: Questionnaire survey. Cross-sectional study.	No detailed information provided.
5	Denison et al., 2008[7]	Ndola, Copperbelt, Zambia	2003	Adolescents 16–19	n = 40	Qualitative study: Semi-structured Interview.	Voluntary counselling and testing (VCT).
6	Kankasa et al., 2009[28]	University Teaching Hospital in Lusaka, Zambia	2006–2007	Children admitted to hospital wards. 0–24 months.	n = 15,670	Quantitative study: Chart review.	HIV counseling and testing.
7	Megazzini et al., 2009[29]	Public sector labor wards in Lusaka.	2005–2006	Women in the first stage of labor and unaware of their HIV status.	n = 217	Quantitative study: Cluster randomized trial.	Rapid HIV testing.
8	Sanjana et al., 2009[30]	Health facilities in Luapula Province and Copperbelt Province.	2007	Lay counsellors, facility manager, counselling supervisor and clients. Lay counsellors: 32–59	Interview: Lay counselors: n = 19 Health facility managers: n = 10 CT health facility clients: n = 95 Focus group: Health care workers: n = 16	Mixed methods. Quantitative: Chart review. Qualitative: Interview. Focus group discussion.	HIV counselling and testing.
9	Kelley et al., 2011[14]	Neighborhoods in Kigali, Rwanda and neighborhoods in Lusaka, Zambia.	2004	Men. 15–60 Women. 15–49	Rwanda: n = 600 Zambia: n = 603	Quantitative study: Questionnaire survey. Cross-sectional study.	Couple voluntary HIV counseling and testing (CVCT).
10	Sikasote et al., 2011[19]	VCT centers mining towns in Copperbelt Province, Zambia.	2007–2008	People who tested for the first time with negative result at VCT. 18–53	Baseline: n = 42 Follow-up: n = 31	Qualitative study: Serial interviews. Focus group discussion.	Voluntary counselling and testing (VCT).
11	Jurgensen et al., 2012[24]	Kapiri Mposhi, a rural district in the Central Province and Lusaka, an urban province in Zambia	2007	Residence in Kapiri Mposhi and Lusaka.	Interviews: n = 17. VCT counsellors interviews: n = 10. Focus group: n = 17.	Qualitative study: In-depth interview. Focus group discussion.	Voluntary counselling and testing (VCT).
12	Wall et al., 2012[38]			Influential network leaders (INL), 36–53 Influential network agent (INA), 29–44 Heterosexual couples, mean age of men = 33 mean age of women = 27	INL n = 68 INA n = 320 Couple n = 1727	Quantitative study: Cluster randomized trial.	Couple voluntary HIV counselling and testing *(*CVCT*)*.
13	Banda, 2013[31]	University Teaching Hospital, Lusaka, Zambia.	2009	Caretakers brought a child to Pediatric department.	n = 239 (women n = 226)	Quantitative study: Questionnaire survey. Cross-sectional study.	No detailed information provided.
14	Fylkesnes et al., 2013[22]	Rural villages in Monze district, Southern province, Zambia.	Baseline: 2009 Intervention: 2010 Follow-up: 2010–2011	Men and women 16+	Baseline: n = 1501 Follow-up: n = 1220	Quantitative study: Cluster randomized trial.	Voluntary HIV counselling and testing (VCT). Home-based.
15	Gari et al., 2013[15]	South and central provinces of Zambia (Chivuna, Mbeza, Mazabuka and Lusaka)		Permanent residents. 18+	n = 1716	Quantitative study: Questionnaire survey. Cross-sectional study.	No detailed information provided.
16	Jurgensen et al., 2013[21]	Rural clusters in Monze district, Southern Province, Zambia.	Baseline: 2009 Follow-up: 2010–2011	Adults. 16+	Baseline survey: n = 1500 Follow-up survey: n = 1107	Quantitative study. Cluster randomized trial.	Voluntary counselling and testing (VCT). Home based.
17	Jurgensen et al., 2013[37]	Clusters in Monze district, Southern Province, Zambia.	Baseline: 2009 Follow-up after intervention: 2010–2011	Adults	Survey: Baseline survey: n = 1501 Follow-up survey: n = 1220. Both surveys: n = 1120 Interview: n = 21 Focus group: n = 6	Mixed Methods. Quantitative: Cluster randomized trial. Qualitative: In-depth interview. Focus group discussion.	Voluntary counselling and testing (VCT). Home based.
18	Musheke, Bond, & Merten, 2013[6]		2010–2011	Couples, women and men, lay counsellor, and nurses. 18+	Couples: n = 10 Women abandoned by spouses: n = 5 Men abandoned by spouses: n = 2 Lay HIV counsellors: n = 5 Antenatal clinic nurses: n = 2	Qualitative study: Open end in-depth interview.	Couple HIV counselling and testing (CVCT).
19	Singh et al., 2013[39]	Kenya, Zambia, and Zimbabwe.	Kenya: 2008–2009 Zambia: 2007 Zimbabwe: 2005–2006	Married or cohabiting women. 15–34	Age 15–24: Kenya n = 1170; Zambia n = 1169; Zimbabwe n = 1648. Age 25–34: Kenya n = 2051; Zambia n = 1880; Zimbabwe n = 2058.	Quantitative study: Questionnaire survey. Cross-sectional study.	No detailed information provided.
20	Brennan et al., 2014[5]	Rural Zambia.	2009–2010	Pregnant women	n = 280	Quantitative study: Chart review.	Rapid saliva-based HIV testing. Home-based.
21	Czaicki et al., 2014[18]	Government clinics in Ndola, Copperbelt, and one mobile testing unit.	First CVCT visit: 2011–2012 Follow-up: before 2012/12	Concordant negative and discordant couple. 16+	n = 10,806 couples.	Quantitative study: Questionnaire survey. Longitudinal study.	Joint voluntary HIV testing and counseling.
22	Denison et al., 2014[20]	Urban township of Chifubu, Ndola, Copperbelt, Zambia.	2004	Adolescent. 16–19	Survey: n = 550 Interview: n = 550	Mixed methods. Quantitative: Questionnaire survey. Cross-sectional study. Qualitative: Interview.	No detailed information provided.
23	Levey and Wang, 2014[32]	VCT service sites in Copperbelt and Luapula, Zambia.	2009	Clients of VCT services. 15+ Health facility managers.	Interview: Clients: n = 400 Facility managers: n = 87	Mixed methods. Qualitative: Interview. Observation (site environment). Quantitative: Chart review.	Voluntary counselling and testing (VCT).
24	Sutcliffe et al., 2014[34]	HIV clinic at Macha Hospital in Choma District in Southern Province, Zambia	2010–2012	Infants.	n = 403	Quantitative study: Chart review.	Infant HIV testing
25	Hensen et al., 2015[26]	Rural and peri-urban area, Lusaka, Zambia.	2011–2012	Men. 15–60	n = 2828	Quantitative study: Cluster randomized trial.	Rapid HIV testing and counselling. Home-based.
26	Hensen et al., 2015[23]	Rural districts, Lusaka Province, Zambia	2013	Men.	n = 2376	Quantitative study: Stepped-wedge cluster randomized trial.	Rapid HIV testing. Home-based.
27	Mwangala et al., 2015[33]	University Teaching Hospital, Lusaka, Zambia.	2013	Lay counselors, nurses and laboratory personnel.	Lay counselors: n = 4 Nurses: n = 4 laboratory scientists: n = 4 laboratory technologists: n = 4	Qualitative study: 16 in-depth interviews. 2 focus group discussion.	Voluntary counselling and testing (VCT).
28	Wang et al. 2015[36]	Livingstone, Monze, and Choma district, Southern Province, Zambia.	Interim immunization data: 2013 Focus groups: 2014–2015	Health facilitates.	Cluster randomized trial: n = 60 Focus group: n = 8	Mixed Methods. Quantitative: Cluster randomized trial. Qualitative: Focus group discussion.	Dry Blood Spot (DBS) testing.
29	Kelley et al., 2016[35]	Kigali, Rwanda and Lusaka, Zambia		Influential network leaders in the faith-based, non-governmental, private, and health sectors. Influential network agents. CVCT clients.	Zambia: INA n = 53 INL n = 31 Client n = 1271 Rwanda: INA n = 33 INL n = 27 Client n = 3895	Quantitative Study: Questionnaire survey. Cross-sectional study.	Couple voluntary HIV counseling and testing (CVCT).
30	Merten et al., 2016[16]	Rural (Mbeza and Chivuna) and urban sites (Lusaka and Mazabuka) in central and southern Zambia.	Qualitative: 2009–2010 Quantitative: 2010–2011	Qualitative: Caregiver of HIV positive children; caregivers living with HIV. Quantitative: caregivers living with children under 15 years old.	Focus group: n = 30 Interview: n = 12 Survey: n = 304	Mixed methods. Qualitative: Interview. Focus group discussion. Quantitative: Questionnaire survey. Cross-sectional study.	No detailed information provided.
31	Musheke et al., 2016[16]	Urban area in Lusaka, Zambia	2010–2011	Marital partners of PLHIV who never tested for HIV. HIV service provider from public sector. Lay HIV counsellors.	Partners of PLHIV: n = 30 Health service provider: n = 10 Lay HIV counsellor: n = 8	Qualitative study: Ethnographic field work. Interview. Focus group discussion.	Voluntary counselling and testing (VCT).
32	Nelson et al., 2016[25]	Urban and rural area of Zambia	2007	Women 15–49	n = 5014	Quantitative study: Questionnaire survey. Cross-sectional study.	ELISA and Western blot test

#### Time of data collection

Twenty-nine studies provided the time of data collection, which ranged from 1995 to 2015. Twenty-seven of the studies reported the results based on data collected between 2002 and 2015, and only two studies [12, 13] used data collected before 2002. Among these twenty-nine studies, most of the data was collected within a year or two, and seven studies [13, 16, 21, 22, 34, 36, 37] used data that took up to three years to collect. For example, Fylkesnes and Siziya [2004] started the baseline data collection in 1996 and follow-up data collection in 1999. In another study led by Fylkesnes [22], the researchers started the baseline data collection in 2009, and conducted the intervention and follow-up data collection in 2010 and 2011.

#### Target population

All of the studies provided information on the target population. The target population included vulnerable group such as pregnant women and infants [4, 5, 29], adolescent and children [7, 20, 28], women [25, 39], and partners of HIV patients [16]. Studies also reported on the programs that focused on men [23, 26], couples [6, 16, 18, 38], general population [12–16, 19, 21, 22, 24, 32, 37], health care workers [6, 16, 27, 30, 32, 33], care givers who live with HIV patients [16, 31], Influential Network Leaders (INL) and Influential Network Agents (INA) [35, 38]. There was also one study that assessed the HIV diagnosis program within the health care service environment [36]. The age of the population was reported in twenty studies. Two of the studies targeted infants and children [28, 34], and the rest of them reported the age of their participants from 15 to 60. The participants were reported at least 15 years old in studies focusing on adults.

#### Types of HIV-testing

Twenty-five studies provided information on the types of HIV testing. All of them were HIV-testing for adults except one for infants [34]. The primary testing applied in the research included: thirteen voluntary HIV counselling and testing (VCT) [7, 12, 13, 16, 19, 21, 22, 24, 28, 30, 32, 33, 37], five HIV counselling and testing targeting couples [6, 14, 18, 35, 38], and three rapid testing [5, 23, 26, 29]. The techniques used in the HIV testing included Dry Blood Spot testing (DBS)[28, 34], ELISA and Western blot testing[25]. Among the studies that reported the HIV testing types, six of them reported home-based testing [5, 21–23, 26, 37].

#### Research methodology

Among the studies, nineteen were quantitative studies [5, 12–15, 18, 21–23, 25–29, 31, 34, 35, 38, 39], six were qualitative studies [6, 7, 16, 19, 24, 33], and seven used mixed methods [4, 16, 20, 30, 32, 36, 37]. The quantitative research methods used in the quantitative studies and mixed methods studies included chart review, questionnaire survey, and intervention studies. Five studies conducted chart review [5, 28, 30, 32, 34]. Twelve studies used questionnaire survey, among which, ten were cross-sectional studies [4, 14–16, 20, 27, 31, 35, 39] and two were longitudinal studies [13, 18]. Nine studies were intervention studies, among which, eight used cluster randomized trial [12, 21, 22, 26, 29, 36–38], and one conduced stepped-wedge cluster randomized trial [23]. The qualitative research methods used in the qualitative studies and mixed methods studies included ethnographic field work, observation, interview, and focus group discussion. One study conducted ethnographic field work [16], one used observation method [32], eleven conducted interview [6, 7, 16, 19, 20, 24, 30, 32, 33, 37], and nine used focus group discussion [4, 16, 19, 24, 30, 33, 36, 37]. Information about sample size was provided in all studies. The sample size of quantitative studies ranged from 60 to 15670. The sample size of qualitative studies ranged from 16 to 95.

### Multi-level factors associated with HIV-testing

Main factors that might influence the uptake of HIV-testing were summarized and categorized into with four socioecological domains including individual level, family level, health infrastructure and health system level, and socio-cultural level (Table 2).

**Table 2:** Main results of the studies.

Author/ Publication year	Main Results			
	Individual factors	Family factors	Healthcare infrastructure and health system factors	Social Cultural factors
Fylkesnes et al., 1999[12]	Testing rates were the lowest among adolescents. HIV-testing rate was positively associated with education attainment, but did not differ regarding geographic location or sexual activity. Urban population, male participants, and people perceived with high risks were more willing to take the test. In rural area, VCT use did not differ by gender, while in urban area, men used the service more than women. For residents aged 25–39, the rural group use the service more frequently than the urban and town residents. Previous test history did not appear to influence the uptake of the tests.			
Chi et al., 2004[27]	Providers who had tested for HIV are more likely to recommend routine testing than those who had never tested (60% vs. 47%, p = 0.05). Providers who correctly estimated the prevalence are more likely to recommend routine testing than those who could not (56% vs. 42%, p = 0.05).		Physicians (OR = 1.9), practioners with research affiliations (OR = 2.3), and practioners in Lusaka (OR = 9.0) were more likely to offer testing. 52% (n = 116) of the participants recommend HIV screening in uncomplicated pregnancies. 100% recommend HIV screening after giving the scenarios. Providers from private facilities are more likely to support routine HIV testing in pregnancy compare to those in district facilities, (75% vs. 47%, p = 0.001).	
Fylkesnes and Siziya, 2004[13]	The testing rate is positively related to the years of education except for two age groups (<8 years vs. >12 years of schooling). 15–24 years of age: OR = 3.4; 95% CI: 1.33–8.83. 25–49 years of age: OR = 2.8; 95% CI: 1.61–4.86. The readiness for VCT was higher in age group 20–24 (49%) than in age group 40–49 (23%). Factors positively associate with readiness for VCT: 15–24 years of age: self-perceived risk of being HIV infected (OR = 1.9; 95% CI: 1.23–2.90). 25–49 years of age: poor self-rated health (OR = 1.9; 95% CI: 1.41–2.43) and previous test experience (OR = 2.2; 95% CI: 1.54–3.25).		The acceptability of VCT varied according to the service delivery: 12% among participants who were offered services at local clinic and 56% among those who were offered at home (RR = 4.7).	
Thierman et al., 2006[4]	Significant demographic factors for taking the HIV testing include: age below 20 (aRR = 1.14), unmarried (aRR = 1.14), first-time pregnant (aRR = 1.14), receiving education less than 7 years (aRR = 1.15), and low income (aRR = 1.14).			
Dension et al., 2008[7]		Negative reaction from family or friends discouraged the participants in seeking of VCT. Participants took VCT often with friends, but rarely with family members.		
Kankasa et al., 2009[28]	Testing rates were significantly associated with age and which hospital ward the children visited. The highest counseling rates were found among children <12 months of age (86.4%) and among admissions to the malnutrition (88.4%) and diarrhea/rehydration (91.5%) wards.			
Megazzini et al., 2009[29]	Testing rate were higher among women who were primigravida than those who were not (aOR = 1.5; 95% CI: 1.1 to 2.1). Test rate were higher among women who were offered VCT than who declined VCT during Antenatal care (ANC) (aOR = 3.7; 95% CI: 2.8 to 5.1).			
Sanjana et al., 2009[30]			Lay counsellors provide up to 70% of VCT services, and their service quality was accepted by facility managers. Data indicated lower error rates for lay counsellors than healthcare workers in VCT registers.	
Kelley et al., 2011[14]	Facilitator to CVCT: to know one’s test result (91%), to plan for the future (35%).	Facilitator to CVCT: to prevent transmission between partners (14%), and to prevent mother-to-child transmission. Barriers for taking the CVCT: partner reaction (24%),	Barrier to CVCT: distance to test facilities and cost (10%).	Barrier to CVCT: stigma (51%).
Sikasote et al., 2011[19]	Factors facilitating the decision-making: susceptibility, identification of risk factors; needs to know their HIV status to regain control of their lives.		Post-test support were needed, including additional information, supportive networks, life-skills training and access to recreational service.	
Jurgensen et al., 2012[24]	Barriers to VCT: fear and burden of knowing their status, stress and detriment to health, concern of losing future opportunity for education, work and marriage.		Barrier to VCT: the concern of confidentiality of VCT facilities.	Barriers to VCT: stigma and discrimination.
Wall et al., 2012[38]	Factors related to uptake of HIV testing: being employed in the sales/service industry (aOR = 1.5; 95% CI: 1.0–2.1) vs. unskilled manual labor; owning a home (aOR = 0.7; 95% CI: 0.6–0.9) vs. not; having tested for HIV with a partner (aOR = 1.4; 95% CI: 1.1–1.7) or along (aOR = 1.3; 95% CI: 1.0–1.6) vs. never having tested; inviting couples (aOR = 1.2; 95% CI: 1.0–1.4) vs. individuals.	Cohabiting couples were more likely to take the testing than non-cohabiting couples (aOR = 1.4; 95% CI: 1.2–1.6).		Significant INA characteristics as predictors of CVCT uptake included promoting in community-based (aOR = 1.3; 95% CI: 1.0–1.8) or health networks (aOR = 1.5; 95% CI: 1.2–2.0) vs. private networks; the woman (aOR = 1.6; 95% CI: 1.2–2.2) or couple (aOR = 1.4; 95% CI: 1.0–1.8) initiating contact vs. INA; couple being socially acquainted with the INA (aOR = 1.6; 95% CI: 1.4–1.9) vs. not; home invitation delivery (aOR = 1.3; 95% CI: 1.1–1.5) vs. in other settings; and easy invitation delivery (aOR = 1.8; 95% CI: 1.4–2.2) vs. difficult distribute.
Banda, 2013[31]	Main reason for not accepting HIV test was fear of death. 69% (n = 165) of the participants were willing to take HIV test for themselves. 99% (n = 239) agreed the hospital provide routine HIV counseling and testing services. 98% were willing to let the siblings of the child take HIV testing.			
Fylkesnes et al., 2013[22]	Knowing HIV status, being reluctant to give blood and having been tested were the main reason for test refusal for participants who accepted counselling. Women and more educated people are more likely to be tested at baseline survey and in the control arm.			
Gari et al., 2013[15]		Determinants for not being tested: disruptive couple relationships (OR200A = 2.48; 95% CI: 1.00–6.19). The influence of unequal power relationships within the couple were underestimated.		Determinants for not being tested: tolerance to gender-based violence (OR = 2.10; 95% CI: 1.05–4.32) and fear of social rejection (OR = 1.48; 95% CI: 1.23–1.80).
Jurgensen et al., 2013[21]			Home-based Voluntary Counselling and Testing have a larger impact on stigma than other testing approaches (β = 0.78, p = 0.080 vs. β = -0.37, p = 0.551).	Association was found between being tested for HIV and reduction in stigma (β = -0.57, p = 0.03).
Jurgensen et al., 2013[37]	Main reasons for accepting HB-VTC: wanted to know status (77%), visited by home-based counsellor (14%), felt at risk (2%). Acceptance of HIV testing is also dependent on gender. Main reasons for not accepting the intervention included prior knowledge of HIV status, no wish to give blood for testing, lack of trust in the counsellors.	Main reasons for accepting HB-VTC: encouraged by partner (2%).	Acceptance of HIV testing is dependent on stigma and trust. Main reasons for high acceptance of HB-VTC are the convenience, confidentiality, credibility of the test, the easy accessibility of counselors, convincing consent process, and encouragement for couple counseling.	
Musheke, Bond, & Merten, 2013[6]		Positive factors associated with testing: notably disclosure of HIV status to marital partner and renewed commitment to marital relationship. Negative factors: abandonment, verbal abuse and cessation of sexual relations.	Positive factor associated with testing: adherence to treatment.	Positive factor associated with testing: formation of new social networks.
Singh et al., 2013[39]	Education was positively associated with testing for both age groups, and the associations were constantly significant for women aged 15–24 years (p<0.01).		The intolerance of gender-based violence was positively associated with testing for women aged 25–34 in all the three countries, the associations were significant in Zambia (among women reporting being tested: OR = 1.24, p<0.10; among women reporting being tested in the past year: OR = 1.29, p<0.05).	
Brennan et al., 2014[5]			44.3% (n = 124) of the 280 participants give birth at home with the assistance of a trained traditional birth attendants (TBAs).	
Czaicki et al., 2014[18]	Significant predictors of follow-up testing included age increase of the man (aOR = 1.02/year) and the woman (aOR = 1.02/year) and either partner being HIV+ (man: aOR = 2.57; women: aOR = 1.89). Predictor of follow-up testing among concordant negative couples is being tested previously (man: aOR = 1.29; couple: aOR = 1.22).		The introduction of a Good Health Package increased follow-up testing among discordant (aOR = 2.93) couples and concordant negative (aOR = 2.06) couples.	
Denison et al., 2014[20]	Factors associated with testing include: having ever had sex (aOR = 6.43; 95% CI: 2.14–19.30] and dropping out-of-school (aOR = 2.95; 95% CI: 1.32–6.59).	Factors associated with testing include: family’s positive attitude for taking an HIV test (aOR = 5.08; 95% CI: 1.16–22.35) and having discussed with a family member about taking an HIV test (aOR = 3.51; 95% CI = 1.08–11.47).		
Levey and Wang, 2014[32]	Women were more likely to use VCT facilities; oldest clients tended to visit private for-profit sites, while younger ones visited NGO sites and private sites. Higher educated clients were more likely to use NGO, while only 6% of the less educated Zambians accessed VCT service.		Private for-profit sectors sometimes over-performed other sectors in HIV testing. Convenience overweighed price as an essential factor for selecting VCT site. There is a serious underperformance across the sectors in counselling about key risk reduction methods. Less than one-third of clients received counselling on reducing number of sex partners and only 5% of clients received counselling on disclosure.	
Sutcliffe et al., 2014[34]			The majority of mothers (80%) and infants (67%) received PMTCT. The total median time from sample collection to return of results to the caregiver was 92 days.	
Hensen et al., 2015[26]	Men aged 20–29 were more likely to accept the testing compared to those aged 15–19 (adjusted prevalence ratio = 1.74; 95% CI: 1.49–1.99, p<0.001). Widowed men were more likely to report ever-testing compared with single men (adjusted prevalence ratio = 1.76; 95% CI: 1.37–2.14, p<0.001). Factors positively associated with acceptance of testing also include receiving secondary/higher education and being married.	Men whose female partner reported testing were more likely to report ever-testing than those whose partner never tested (adjusted prevalence ratio = 1.59; 95% CI: 1.27–1.90, p<0.001).		
Hensen et al., 2015[23]	Multiple-testers were positively associated with age (30–39), higher levels of education, being employed, and availability of ART in testing sites on the day of the audit. Acceptance of home-based testing was similar among ever-tester and multiple-tester (adjusted prevalence ratio = 1.05; 95% CI: 0.93–1.17). Acceptance was lower among men over 40 years relative to men in the 20–29 age group (adjusted prevalence ratio = 0.76; 95% CI: 0.65–0.87) Little evidence showed that acceptance of home-based testing was associated with occupation, education, religion or marital status.	Participants (n = 719) were more likely to take multiple testing if their spouse reported ever-testing (adjusted prevalence ratio = 3.02 95% CI: 1.37–4.66).		
Mwangala et al., 2015[33]			Confidentiality and privacy were greatly compromised due to limited space. Difficulties in upholding consent were reported in provider-initiated testing in in-patient settings. Key factors impacting on quality of testing: non-adherence to testing procedures, high workload, and inadequate training and supervision. Lay counselors reported difficulties in finger pricking and obtaining adequate volumes of specimen; non-laboratory providers had problems in interpreting invalid, false-negative and false-positive results.	
Wang et al. 2015[36]			The Simple Intervention has 16.6% (90% CI: -7%- 46%, p = 0.26) greater change in average monthly testing than the controlled group, the Comprehensive Intervention has a 10% (90% CI: -10%-36%, p = 0.43) greater change. The Simple Intervention resulted in a greater change of 15.76 (90% CI: 7.12–24.21, p<0.01) in total maternal re-tests over baseline than the controlled group, the Comprehensive Intervention has an impact of 10.93 (90% CI: 1.52–20.33, p = 0.06)	
Kelly et al., 2016[35]			77% INAs and 100% INLs in Zambia reported promoting CVCT via group forums. 79% INAs and 81%INLs in Zambia reported promoting CVCT via speaking to a community leader in the past month.	
Merten et al., 2016[16]	Main reason for letting children take HIV testing: poor health of children (OR = 0.23; 95% CI: 0.11–0.51] and suspicions of HIV infection as the underlying cause (58.7%).	Main reasons for not letting children take HIV testing: fears of the reactions from the family (28%); to be considered HIV+ oneself (22%); a disagreeing spouse (20%); and having no idea where to take the test (12%).		Main reasons for not letting children take HIV testing: men’s decision power, economic dependency on husband, concerns for reputation, stigma, fear of HIV-related discrimination (OR = 1.35; 95% CI: 1.04–1.74), and observed stigmatization of HIV positive children in neighborhood (aOR = 1.69; 95% CI: 1.20–2.39).
Musheke et al., 2016[16]	Reasons for non-uptake HIV testing: good physical health conditions, perception of being infected, psychological burden of living with HIV (e.g. knowledge such as HIV-positive status led to rapid physical deterioration of death), lack of self-efficacy (perceived inability to sustain uptake of life-long treatment), and self-stigma.	Reasons for non-uptake HIV testing: fear of being blamed by marital partner	Reasons for non-uptake HIV testing: alternative treatment for HIV symptoms.	
Nelson et al., 2016[25]		Significant association was reported between intimate partner violence and HIV testing in rural areas only (OR = 1.17; 95% CI: 1.02–1.34).		

#### Individual level

Demographic (e.g., gender, age, marital status, etc.) and socio-economical characteristics (e.g., income, urban/rural residence, education attainment, etc.) affects people’s seeking and accessing health services. Decision-making and practices related to HIV-testing also could be influenced by knowledge of HIV/AIDS, perceived risk of HIV infections, attitudes and perceptions of HIV-testing service, and previous history and experiences of HIV-testing. Existing literature suggest complicated associations between individual factors and HIV-testing practices depending on target populations and the specific approach of HIV-testing services.

A few studies showed that higher education attainment is significantly associated with higher acceptability and higher rate of HIV-testing [12, 13, 22, 26, 32, 39]. Similarly, higher socio-economic status was positively associated with HIV-testing. For example, having a job was positively related to taking HIV-testing multiple times [23]. Individuals who worked in sales or the service industry were more likely to take HIV-testing compared to unskilled manual labors (aOR = 1.5, 95% CI = 1.0–2.1) [38]. One recent study reported no significant association between education or occupation and acceptance of home-based HIV-testing [23]. Another exception was reported in a study among women who visited antenatal clinics, which indicated that lower education level (aRR = 1.15) and lower income (aRR = 1.14) was associated with uptake of HIV-testing [4].

Mixed results have been found regarding how gender, age, and marital and pregnancy status associated with HIV-testing practices. Earlier studies suggested that men were more willing to take HIV-testing and reported a higher proportion of previous testing than women [12]. Two recent studies indicated that women were more likely to use VCT facilities [22, 32]. The readiness for HIV-testing service was higher in younger adults (49% for age group 20–24 vs. 23% for age group 40–49) [13]. Younger men (aged 20–29) also reported higher acceptance of home-based HIV-testing compared to men older than 40 years of age [23]. However, one study on a couple testing project indicated that older couples were more likely to take follow-up testing [18]. One study with a focus on HIV-testing among men, reported that men aged 20–29 were more likely to accept HIV-testing compared to those aged 15–19 (aRR = 1.74, 95% CI = 1.49–1.99, p < .001) [26]. This result was in line with a previous study reporting that the HIV-testing rate was lowest among adolescents [12]. According to a study conducted among antenatal attendees aged 16 to 46 in Lusaka, women younger than 20 years old were more likely to accept HIV-testing service [4].

For men, being married was positively associated with acceptance of testing [26]. Widowed men were more likely to report having experiences of HIV-testing compared with single men (aRR = 1.76, 95% CI = 1.37–2.14, p < .001). For women, being unmarried was associated with acceptance of HIV-testing service in antenatal care clinics [4]. Women pregnant for the first time were more likely to undergo HIV-testing [4, 29].

Attitudes, perceptions, and previous experiences regarding health risks, health status, and HIV/AIDS play a critical role in decision-making and uptake of HIV-testing among Zambians either serving as facilitators or barriers. For example, susceptibility of HIV infection, identification of high-risk behaviors, desire to know HIV serostatus to plan for their future and control their life, could motivate people to accept and take HIV-testing service [12, 14, 19, 37]. People who had previous testing experience with negative HIV-serostatus were more likely to accept and take HIV-testing again [18, 29, 38].

Results show that the uptake of HIV-testing services is also related to the factors in cognitive level. People who thought they were in good health with low susceptibility of HIV infection were less likely to intend to take a testing [16]. A number of studies, particularly qualitative studies, indicated that people refused HIV-testing because of their perceptions of already being infected and the fear of HIV infections [16]. Fears included the fear of deteriorated health and death, fear of the psychological burden (e.g., stress) resulted from knowing HIV status, fear of social rejection, and concerns and worries of losing future opportunities for education, job and marriage [15, 16, 24]. Main barriers also include self-stigma related HIV and lack of self-efficacy to sustain a life-long treatment [16]. Being reluctant to give blood during the testing could be another reason for refusal of HIV-testing service [22, 37].

It is notable that different demographic factors may interact with each other, and the attitudes and perceptions factors vary across diverse sub-groups. For example, Fylkesnes and the colleagues reported that the use of HIV-testing service did not differ by gender in rural areas, but in urban areas, men used testing services more than women [12]. In addition, although urban population reported higher rate of HIV-testing rates than rural population, for residents aged 25–39, the rural residents use HIV-testing service more than their counterparts living in urban and town [12]. Their study conducted in 2004 [13] suggested that for young adults (15–24 years of age), self-perceived risks of HIV infection was positively associated with their willingness to get HIV-testing (OR = 1.9, 95% CI = 1.23–2.90). Among older group (25–49 years of age), the readiness of taking HIV-testing was associated with poor self-rated health status (OR = 1.9, 95% CI = 1.41–2.43) and previous testing experience (OR = 2.2, 95% CI = 1.54–3.25).

Along with the national-level expansion of HIV-testing services, an increasing number of empirical studies explore the characteristics of pediatric and adolescents HIV-testing practices. Kankasa and colleagues discovered that HIV-testing rate for young children was significantly associated with child’s age and the type of hospital wards the children visited [28]. The highest counseling and testing rate was found among children younger than 12 months of age (86.4%) and among admission to the malnutrition (88.4%) and diarrhea/rehydration wards (91.5%) [28]. Facilitators for pediatric HIV-testing included poor health status of children and suspicions of HIV infection [16]. For adolescents, the ones who had initiated sexual activity (aOR = 6.43, 95% CI = 2.14–19.30) and had dropped out of school (aOR = 2.95, 95% CI = 1.32–6.59) were more likely to take HIV-testing [20].

#### Family level

Partner relationship, partners’ experiences and attitudes towards HIV-testing, expected reactions of family, and perceptions of potential consequences following HIV-testing critically affected the process of decision-making and the post-diagnosis adaptation. Family relationship and contexts also dominated the uptake of pediatric HIV-testing and shape the adolescents’ decision and actions given that children were dependent of their caregivers in most of aspects in their life. A quantitative study among 603 households in Lusaka about their knowledge and perceptions of couples voluntary counseling and testing (CVCT) suggested that main reasons of accepting CVCT included the desires to prevent HIV transmission between partners and prevent mother-to-child transmission [14]. The relationship with partners and the living arrangement of couples could also affect the decision-making process regarding HIV-testing. Cohabiting couples were more likely to take the CVCT compared to non-cohabiting ones (aOR = 1.4, 95% CI = 1.2–1.6) [38]. Disruptive couple relationship was a key determinate for declining HIV-testing (OR = 2.48, 95% CI = 1.00–6.19) [15].

Partners’ experiences of HIV-testing and their attitudes towards HIV-testing service could be significantly associated with the uptake of HIV-testing. For example, men whose female partner reported previous HIV-testing were more likely to report ever-testing compared to those whose partner had never got tested (aRR = 1.59, 95% CI = 1.27–1.90, p < .001) [26]. Couples who were invited together in HIV-testing service were more likely to take HIV-testing than those who were invited individually (aOR = 1.2, 95% CI = 1.0–1.4) [38]. A quantitative study among home-based HIV-testing suggested that people were more likely to take multiple testing if their partners reported previous HIV-testing [23]. Partners’ encouragement was also listed as main reason for accepting home-based HIV-testing [37].

Perceived consequences of HIV diagnosis could be either a facilitator or barrier for uptake of HIV-testing. The various perceptions were rooted in the uncertainties in knowing and disclosing HIV positive serostatus. One qualitative study that explored couple experiences of provider-initiated couple HIV-testing in antenatal clinics demonstrated both positive and negative outcomes following HIV-testing [6]. Couple testing might positively result in disclosure of HIV status to partners, renewed commitment to marriage, access and adherence to treatment, and the development of new social networks, but on the other hand, HIV-infected individuals might negatively face abandonment, verbal abuse and cessation of sexual relations [6]. Perceived negative reactions from partners halted them from using HIV-testing service [14]. In a qualitative study among marital partners of HIV-infected persons, most of the participants believed that the chance of being in a HIV-discordant relationship was so small that they viewed HIV-testing as unnecessary [16]. In addition, declining HIV-testing was used to avoid blame or accusations of being responsible for HIV-infection, so they could “maintain moral credibility in the marital relationship” [16].

Adolescents’ HIV-testing and pediatric HIV-testing were greatly influenced by their family context. Perceived negative reactions from family or friends discouraged the youth in seeking VCT service [7]. A study conducted among adolescents aged between 16 and 19 years indicated that participants who had discussed HIV-testing with a family member were more likely to underdo HIV-testing (aOR = 5.08, 95%CI = 1.16–22.35) [20]. Family positive attitude towards HIV-testing service was also a significant facilitator for adolescents’ uptake of testing (aOR = 3.51, 95% CI = 1.08–11.47) [20]. A recent pediatric HIV-testing study identified many barriers related to family relationships including fears of reactions from the family, disagreement with spouse on HIV-testing issues, economic dependency on husband, and men’s dominating power in decision-making [16].

#### Health infrastructure and health system level

The types and characteristics of health facilities were directly related to whether or not practitioners provided HIV-testing services to their clients. An early study conducted among maternity-based health care providers throughout Zambia indicated physicians (OR = 1.9), health care providers with research affiliations (OR = 2.3), and those located in Lusaka (OR = 9.0) were more likely to provide HIV-testing services [27]. Only 52% of the respondents believed that HIV screenings should be routinely provided to women uncomplicated pregnancies. Health care providers from district facilities (OR = 2.8), from Lusaka (OR = 10.1), and from research facilities (OR = 3.4) were more likely to prescribe ART routinely to reduce mother-to-child transmission (OR = 3.4) [27]. Lack of availability of ART was the main barrier of prescribing ART [27]. A review of patterns of clients in VCT service reported that women were more likely to use VCT facilities [32]. Younger clients tended to visit NGO sites and private sites, while the oldest age group visited private for-profit sites. Clients with higher education attainment were more likely to use NGO services [32]. Convenience overweighed expenses as an essential factor for people’s selecting VCT site [32]. Distance to the testing facilities and cost were also reported as barriers for access to CVCT service [14].

Quality of HIV-testing service, specific strategies and approach of service delivery, and the relationship between clients and health care providers are critical components shaping people’s selection and experiences regarding HIV-testing. According to Levey and Wang’s review on HIV-testing service across Zambia, underperformance was a serious concern across the sectors in counselling about basic risk reduction methods [32]. For example, less than one-third of clients received advice on reducing the number of sexual partners, and only 5% of clients received counselling on disclosure of their status [32]. In addition, post-diagnosis support were not sufficiently integrated in the existing VCT service, such support could include additional HIV-related information, supportive networks, life-skills training and access to recreational service [19]. Delays in processing and communicating test results were identified in early infant HIV diagnosis in rural Zambia [34]. Based on chart reviews conducted from 2010–2012 for early infant HIV-testing at Macha Hospital, the total median time from sample collection to return diagnosis results to caregivers was 92 days (Sutcliffe et al., 2014). In resource-constrained settings, confidentiality and privacy required in VCT service were greatly compromised due to limited space [33]. Under the circumstance of in-patient setting, there were difficulties in upholding consent in delivering provider-initiated service [33]. Some people were reluctant to take HIV-testing themselves or let their children to get a diagnosis because of their concerns and worries about confidentiality of VCT facilities and potentially ruined reputations [16, 24, 37].

The strategies and approach of delivering HIV-testing service have been evolving in Zambia. The innovative and appropriately adapted ways of providing HIV-testing service have facilitated the expansion of HIV-testing coverage. Czaicki and the colleagues initiated a follow-up of couples’ voluntary HIV counseling and testing (CVCT) service for discordant and concordant negative couples in Copperbelt province. This intervention increased follow-up testing among discordant (aOR = 2.93) and concordant negative (aOR = 2.06) couples. As for pediatric HIV-testing in-patient setting, the majority of parents accepted routine HIV counseling and testing services provided by hospitals and they were willing to let the siblings of the child take HIV-testing [31]. In addition, integrating early infant HIV diagnosis with the expanded program on immunization in rural Zambia showed desirable outcomes. The comprehensive intervention group (provided with the resupply of HIV-testing commodities when necessary and on-site operational support) reported more maternal re-tests over baseline compared to the control group (OR = 10.93, 90% CI = 1.52, 20.33, p = .06) [36]. Empirical studies indicated that home-based VCT were more acceptable compared to VCT provided at local clinic (RR = 4.7) [13]. Home-based VCT service could reduce the negative effect of HIV-related stigma than other testing approaches [21].

In the implementation of task-shifting strategy in providing HIV-testing service, paraprofessionals and community leaders have been positively engaged, and recruited and trained. Their evolvements multiplied the formats of services, and generally promoted the use of HIV-testing services. Lay counselors provided up to 70% of VCT services, and facility managers accepted the quality of their service. Lay counsellors had lower error rates in VCT registers than health care workers [30]. There was also empirical evidence supporting the positive role of influential network leaders (INLs) and influential network agents (INAs) in promoting CVCT in Zambia. For example, in one survey conducted among 3895 clients in Lusaka, 71% of the clients reported hearing about CVCT service from INAs during a one-on-one promotion [35]. In a cohort study over 18 months, 68 INL identified 320 INAs who delivered 29229 CVCT invitation to heterosexual couples resulting in 1727 couple testing (6% success rate) [38]. In the context of reducing mother-to-child transmission, traditional birth attendants (TBAs) could perform rapid saliva-based HIV testing in home and administer single-dose nevirapine in tablet to the mother at labor and syrup to the infant after birth [5]. A feasibility study reported that 93.5% of the participants who gave birth at home with TBAs accepted a rapid HIV test. For HIV-positive women, 81.3% of them took single-dose nevirapine administrated by a TBA within 24 hours prior to birth and 100% of exposed newborns received nevirapine syrup within 72 hours after birth [5].

The relationship between counselors and potential clients could be a key element for the success of promoting HIV-testing in Zambia, especially in the resource-constrained communities. Trusting the counselors was one of elemental factors for taking home-based HIV-testing [37]. High acceptance of the home-based HIV-testing approach was also attributed to the high confidentiality, high credibility of the test, convincing consent process, and easy accessibility of counselors [37]. In the CVCT testing invited by Influential Network Agents (INAs), couples who were socially acquainted with the INAs were more likely to use CVCT services (aOR = 1.6, 95% CI = 1.4–1.9) [38].

#### Socio-cultural level

Of the empirical studies finally included in our review, two quantitative studies focus on the associations between gender inequity and the uptake of HIV-testing. Using data of Demographic and Health Surveys (DHSs) conducted in Zambia (2007), Kenya (2008–2009), and Zimbabwe (2005–2006), Singh and the colleagues examined how education and gender inequality affected the use of HIV-testing service among married women (2013). Their findings indicated that education had a positive relationship with testing for women, especially for young women aged 15–24 years (p < .01). The intolerance of gender-based violence was significantly and positively associated with HIV-testing for women aged 25–34 years in Zambia (among women reporting ever being tested: OR = 1.24, p < .01; and among women reporting being tested in the past year: OR = 1.29, p < .05). The other study based on a cross-sectional study of 1716 randomly selected individuals in the South and Central provinces of Zambia reported similar results [15]. The tolerance to gender-based violence in the household was one of significant barriers for being tested for both men and women (aOR = 2.10, 95% CI = 1.05–4.32).

HIV-related stigma is constantly one of strongest barriers to the uptake of HIV-testing [14, 21, 24, 37]. In a cross-sectional household survey about CVCT conducted among adults in Zambia (n = 603) and Rwanda (n = 600), participants from Zambia reported stigma as the major obstacle to CVCT (51% vs 29% in Rwanda) [14]. Main reasons for not taking children to HIV-testing also included fear of HIV-related discrimination (OR = 1.35, 95% CI = 1.04–1.74) and observed stigmatization of HIV-infected children in neighborhood (OR = 1.69, 95%CI = 1.20–2.39) [16].

One qualitative study with a focus on HIV-testing decisions among both rural and urban districts suggested that HIV-related stigma and discrimination interplayed with the manners of service and the memories of suffering and death among AIDS patients over the last few decades [24]. In-depth interviews revealed the arrangement of the VCT facilities compromised confidentiality, being located in a separate building or a particular room where VCT clinic was clearly visible. Visitors were worried about being seen at or leaving the VCT clinic. Deep fears of knowing HIV status were rooted from their long-term experiences with HIV/AIDS, even though the interviewees knew HIV/AIDS was no longer an incurable fatal disease. The immense social and physical suffering they had been seen among AIDS patients was perceived to be so detrimental to their health that they would rather not know their HIV status. Non-uptake of HIV-testing thus could be viewed as a form of label-avoidance and strong expressions and echoes of memories regarding HIV/AIDS over the last decade [24].

In addition, the embodied memories of non-curable AIDS patients, traditional health beliefs and practitioners as well as religions might also play a complicated role in decision-making regarding uptake of HIV-testing. In Zambia, people may turn to various health facilities when they have health issues, such as public health center, NGOs’ service sites, and privately owned clinics and drug stores [16]. Healthcare providers include herbalists, traditional practitioners, and faith healers [16]. Some of the practitioners advertise their ability to “cure” HIV and other sexually transmitted infections [16]. Some Christianity churches provide faith healing sessions for people suffering health problems including HIV [16]. As an alternative to HIV-testing or ART treatment, people seek herbal remedies and conventional non-HIV medication to mitigate HIV-related symptoms. Some turned to faith healing instead of seeking HIV-testing [16]. They acknowledged the power of prayer and faith in God in dealing with health issues including incurable diseases such as HIV/AIDS [16]. Only when they noticed a declined physical health condition and that other alternative forms of care had become ineffective were they willing to take a test [16].

## Discussion

The rapid growth of empirical studies on HIV-testing in Zambia since 1999 reflects the scale-up of HIV-testing service and national level efforts in combating HIV/AIDS epidemic in Zambia. Along with evolving practices of promoting HIV-testing, the existing literature demonstrates five characteristics. The first is that geographic distributions of the study sites have expanded from urban to rural areas, from Lusaka to other provinces. The second characteristic is that study settings have turned into various formats with the increasing engagement of local communities. The third characteristic is that target populations covered by the studies have become diverse in gender, age and social roles. The forth characteristic shows that, the approaches of HIV-testing service have been diversified adapting to various clients and settings with applications of novel testing techniques. Finally, the past decade witnessed a growing number of longitudinal studies and intervention studies.

Although existing literature has cumulated enormous findings regarding HIV-testing practices in Zambia, to the best of our knowledge, this is the first systematic review of multi-level factors that have affected the uptake of HIV-testing in Zambia. Our review identified some common factors across various populations in different settings. In the individual level, higher education attainment was a strong and constant facilitator across various populations, which is accordance with positive role of education in promoting general health status. Misconceptions of HIV-testing and the emotional burden of knowing one’s HIV diagnosis results are prominent barriers to using HIV-testing services. Coping skills such as how to handle and control fears regarding HIV-testing could be integrated into HIV education and social mobilization activities. One of the fears toward HIV-testing has been the uncertain and perceived negative consequences in social relationships. Marital dynamics, partner relationship, and the relationship with healthcare providers of HIV-testing service greatly affect the decision-making process among adults, especially in the setting of couple testing and counseling. Encourage and support from family members also promote adolescents’ HIV-testing. In terms of health facilities, poor health infrastructure and unsatisfied quality of services hinder people from accessing and using HIV-testing services. Task-shifting strategies and home-based HIV-testing is generally highly accepted by local people, and has promising approaches to increase the rate of HIV-testing when it is appropriately implemented and supervised. As for socio-cultural level factors, HIV-related stigma and discrimination is a root cause for reluctance and struggles in the uptake of HIV-testing for almost all the populations.

Our literature review also examined particular factors for different populations. For example, gender inequality and family power dynamics placed women in vulnerable positions in selecting HIV-testing service and in facing the consequences of knowing and disclosing results of HIV diagnosis. In pediatrics HIV-testing, poor health infrastructure, such as the lack of lab and lab technicians, transportation issues, and insufficient HIV-testing kits, hindered valid and rapid HIV-testing. For couples, the HIV serostatus of partners and partners’ experiences of HIV-testing are significant predictors of using HIV-testing services including couple testing.

In the interpretation and generalization of these results, we need be cautious about several issues. First, the data collection time of the reviewed studies ranges as wide as 20 years. The practices of HIV-testing have been always evolving, and the so have the culture transformation and socio-economic development. Therefore, we need to pay special attentions to the potential differences in study settings at different times. Second, we found conflicting results in the associations between several individual level factors and the uptake of HIV-testing. These associations may be vary across diverse populations and even be changeable within the same population given different specific settings and approaches of HIV-testing. It is not valid to generalize the results without carefully identifying populations or settings. Third, in this review, we only examined the factors related to acceptability of innovative HIV-testing approaches (e.g., home-based VCT). The review and comparison of intervention efficacy go beyond the goals and scope of this review. Forth, although we categorized the factors into different levels, there was no strict cut-off line between different levels. For example, education could be viewed as socio-economic variable in the category of individual level factors, while for women, it could also be an opportunity of empowerment. Education, as an indicator of gender equity, could be in the category of socio-cultural level factors. In addition, we need to pay attention to the interactions between factors at different levels through a socioecological perspective. For instance, individual fears of HIV-testing have been caused by feelings of HIV-related stigma and discrimination.

Although limited by these issues, our findings have several implications in the practices of HIV-testing promotion in Zambia. Individualized strategies and comprehensive services are needed for diverse populations. Our study shows that demographic characteristics affect the decision-making related to HIV-testing use as well as the specific needs and selections of health services. Individual perceptions and experiences also vary by sub-populations. We need to develop creative and flexible approaches to meet increasing individualized needs. The advanced development of HIV-testing techniques has provided a solid ground. On the other hand, numerous studies have called for comprehensive counselling and services beyond sole HIV diagnosis. For example, educations of risk reduction, assistance of HIV-disclosure, and psychological counseling services have not yet been sufficiently covered in the current services [19, 32].

Second, family-based education and intervention, could be strengthened by integrating efforts of improving gender equity in Zambia. While most studies on gender inequity and HIV/AIDS focused on the associations between vulnerability to HIV infection and gender-power inequity[40–42], our findings show fears rooted in gender-power inequity could be an overarching barrier for uptake of HIV-testing among women. In addition to strategies of provider-initiated and home-based HIV-testing, it is necessary to develop family-based intervention to assist couples, particularly serodiscordant couples to set up positive partner relationship to increase HIV-testing rates and improve post-diagnosis adaptation. It is also crucial to respect individuals’ wills when engaging men in such interventions. A recent qualitative study reported healthcare providers’ coercive and subtle strategies to enlist women’s spouses for couple HIV testing [6]. These strategies resulted in men’s feelings of being “trapped” or “forced” to test as part of their paternal responsibility. They also violated the trust relationship between clients and healthcare providers.

Third, capacity building for healthcare providers, especially for paraprofessional HIV-testing counselors, is critical to effectively implementing task-shifting strategies. Engaging trained paraprofessionals into the HIV treatment cascade could be a cost-effective strategy in resource-constrained settings in sub-Saharan Africa. Generally, lay counselors are welcomed by health facility managers and are accepted by clients [30]. A study based on national HIV proficiency testing exercise in 2010 reported overall accuracy of rapid HIV-testing as 97% (95% CI = 96.1–97.8) with no significant difference between laboratory and non-laboratory personnel [9]. However, lay counselors had more difficulties in finger pricking, obtaining adequate volumes of specimen, and interpreting diagnosis results [9, 33]. Key issues impacting quality of services include non-adherence to testing procedures, high workload, and inadequate training and supervision [9]. To improve accuracy of HIV-testing, paraprofessionals should receive more standardized HIV rapid testing training and daily supervision.

Fourth, social facilitators and barriers for improving quality of HIV-testing services should be paid attention to in the health practices. For example, HIV-related stigma and discrimination is still persistent and overwhelming in Zambia. Although along with the widely use of ART treatment, people have realized that HIV/AIDS can be manageable as a type of chronic disease, the collective memories and stories of AIDS patients in the past several decades have been so painful that the knowledge burden of HIV diagnosis may overweigh the benefits of early treatment [16, 43]. In addition, social support is found to be an important facilitator for the linkage to HIV treatment cascade [44, 45]. These factors need to be addressed in social mobilization strategies for HIV-testing promotion [44, 46]. Health facilities can also modify their arrangement of schedule, site location, and environment of HIV-testing clinics to improve their convenience, confidentiality and accessibility.

Several implications for future studies emerged from this review. First, existing studies concentrate in Lusaka. Although it is partly due to high HIV prevalence in Lusaka, it still brings a big concern on generalizability of the findings in these studies. Future research in Zambia need to cover diverse regions. Second, although existing studies have targeted various populations, there is a dearth of data in two high-risk populations, the high-risk populations are prison populations and men who have sex with men. One recent HIV screening study in 6 prisons in Zambia reported overall prevalence of HIV infection as 22.9% among inmates, which was nearly twice the Zambian national estimate [47]. One recent study estimated a 33% HIV prevalence rate among MSM in Zambia [48]. According to the latest Zambia National AIDS Council report, MSM make up about 10% of new HIV infections in Zambia. However, so far there is no systematically collected data of the HIV epidemic among MSM primarily because homosexual behavior is illegal and punishable by the current laws in Zambia. Explanatory studies and preliminary data are needed to develop efficacious projects to conduct and promote HIV-testing in these two vulnerable and marginalized populations. Third, none of the existing empirical studies has tackled the feasibility and acceptability of applying information and communication techniques (ICT) such as website and mobile phone application to improve HIV-testing services. The features of ICT include information exchange in a timely, anonymously, and customized manner, and remote access in areas with limited infrastructures [49]. The advancement of ICT may innovatively address the barriers to the uptake of HIV-testing [50]. Previous studies have suggested the efficacy of applying ICT in HIV testing and counselling services [51–54]. The high accessibility of mobile phones in Zambia and other sub-Saharan African can enable ICT to be effectively utilized in HIV-testing interventions. Fourth, the proportion of longitudinal studies has been low. In addition, most of existing longitudinal studies focused on intervention. We need more longitudinal studies to investigate the complicated interactions between factors at various levels. Some studies rely on chart review, clinic observations and demographic household surveys (DHS) as data resources. Future quantitative studies should use more comprehensive and accurate measures based on appropriate theoretical frameworks to explore the practices of HIV-testing and factors affecting uptake of HIV-testing.

HIV-testing is the first step of HIV treatment cascade. A rapid, accurate and comprehensive HIV testing and counseling service can contribute to HIV-infected persons’ engagement in treatment. With synthesizing multi-level facilitators and barriers for uptake of HIV-testing, our review has provided a summary of implications in public health practices and suggestions for future research directions. We hope the findings based on Zambia can also shed insights on HIV-testing studies in other countries in sub-Saharan Africa.

## Supporting information

S1 File PRISMA checklist. (PDF)
